# One Health contributions towards more effective and equitable approaches to health in low- and middle-income countries

**DOI:** 10.1098/rstb.2016.0168

**Published:** 2017-06-05

**Authors:** S. Cleaveland, J. Sharp, B. Abela-Ridder, K. J. Allan, J. Buza, J. A. Crump, A. Davis, V. J. Del Rio Vilas, W. A. de Glanville, R. R. Kazwala, T. Kibona, F. J. Lankester, A. Lugelo, B. T. Mmbaga, M. P. Rubach, E. S. Swai, L. Waldman, D. T. Haydon, K. Hampson, J. E. B. Halliday

**Affiliations:** 1 Boyd Orr Centre for Population and Ecosystem Health, Institute of Biodiversity, Animal Health and Comparative Medicine, and; 2 School of Geographical and Earth Sciences, University of Glasgow, Glasgow G12 8QQ, UK; 3 Department for the Control of Neglected Tropical Diseases, World Health Organization, Avenue Appia 20, 1211 Geneva 27, Switzerland; 4 School of Life Sciences and Bioengineering, Nelson Mandela African Institution of Science and Technology, PO Box 447, Arusha, Tanzania; 5 Centre for International Health, University of Otago, PO Box 56, Dunedin 9054, New Zealand; 6 School of Veterinary Medicine, University of Surrey, Guildford GU2 7XH, UK; 7 College of Veterinary Medicine and Medical Sciences, Sokoine University of Agriculture, PO Box 3105, Morogoro, Tanzania; 8 Paul G. Allen School for Global Animal Health, Washington State University, Pullman, WA 99164, USA; 9 Kilimanjaro Clinical Research Institute, Kilimanjaro Christian Medical Centre, PO Box 2236, Moshi, Tanzania; 10 Division of Infectious Diseases, Duke University Medical Center, Durham, NC 27710, USA; 11 Ministry of Agriculture, Livestock and Fisheries, PO Box 9152, Dar es Salaam, Tanzania; 12 Institute for Development Studies, Library Road, Brighton BN1 9RE, UK

**Keywords:** One Health, zoonoses, health equity, global health, sustainable development, poverty

## Abstract

Emerging zoonoses with pandemic potential are a stated priority for the global health security agenda, but endemic zoonoses also have a major societal impact in low-resource settings. Although many endemic zoonoses can be treated, timely diagnosis and appropriate clinical management of human cases is often challenging. Preventive ‘One Health’ interventions, e.g. interventions in animal populations that generate human health benefits, may provide a useful approach to overcoming some of these challenges. Effective strategies, such as animal vaccination, already exist for the prevention, control and elimination of many endemic zoonoses, including rabies, and several livestock zoonoses (e.g. brucellosis, leptospirosis, Q fever) that are important causes of human febrile illness and livestock productivity losses in low- and middle-income countries. We make the case that, for these diseases, One Health interventions have the potential to be more effective and generate more equitable benefits for human health and livelihoods, particularly in rural areas, than approaches that rely exclusively on treatment of human cases. We hypothesize that applying One Health interventions to tackle these health challenges will help to build trust, community engagement and cross-sectoral collaboration, which will in turn strengthen the capacity of fragile health systems to respond to the threat of emerging zoonoses and other future health challenges. One Health interventions thus have the potential to align the ongoing needs of disadvantaged communities with the concerns of the broader global community, providing a pragmatic and equitable approach to meeting the global goals for sustainable development and supporting the global health security agenda.

This article is part of the themed issue ‘One Health for a changing world: zoonoses, ecosystems and human well-being’.

## 1. Introduction

While outbreaks of emerging zoonoses, such as Ebola virus disease, galvanize the world's attention, it is the endemic zoonoses that still inflict the much greater burden of mortality and morbidity. In a recent review exploring the associations between zoonoses and poverty, a ranking of ‘important’ zoonoses was made on the basis of human mortality, human morbidity, impact on the livestock sector, amenability to agriculture-based control, and emergence or severity of disease in people [1]. The 13 top-ranked zoonoses were responsible for 2.2 million human deaths and 2.4 billion cases of illness every year [1]. Notwithstanding the devastating impacts of the recent West African Ebola disease epidemic, it is salutary to note that, every year, rabies and leptospirosis are estimated to cause five times as many human deaths, with an estimated 59 000 people dying from each of these diseases annually [2,3]. Other endemic zoonoses may well have impacts of comparable magnitude [1], but in many cases, we lack the data to define and demonstrate these impacts fully.

There are several reasons why, despite causing relatively high mortality and morbidity, endemic zoonoses do not trigger as much international concern as emerging zoonotic diseases, such as those caused by Ebola virus, highly pathogenic avian influenza virus or severe acute respiratory syndrome-coronavirus. First, for most endemic zoonoses, there is little potential for sustained transmission in human populations and therefore little risk of transboundary spread to high-income countries through human movements and contacts. Second, measures for the prevention, treatment and control of endemic zoonoses are often available to protect people and animals in high-income countries and the disease burden is much less substantial than in neglected communities. These factors reduce immediate awareness and concern about disease risk at international level, which in turn impacts on the perceived need to prioritize investments for disease control and prevention in low-income settings.

This lack of prioritization is further exacerbated by problems of disease visibility. Many endemic zoonoses present with non-specific clinical signs in both people and animals and are easy to misdiagnose on clinical grounds [4]. These zoonoses are poorly recognized by healthcare providers [5,6] and are often overlooked in differential diagnoses. Well-validated point-of-care diagnostic tests are rarely available, and diagnosis of chronic stages or sequelae of infections is often difficult. The pattern of under-recognition of zoonoses persists, despite a growing body of evidence that many zoonoses are important causes of common human disease syndromes, such as undifferentiated fever, in both Africa and Asia [6–12].

While several endemic zoonoses have been termed ‘neglected’ [13], the issue of neglect arises not as a result of lack of recognition of or research on the pathogens *per se*. Most of these zoonoses have long been recognized in the medical and veterinary literature, are well understood and are often well controlled in high-income countries. Instead, their neglect occurs because the risks and burden of these zoonoses fall heavily on disadvantaged and vulnerable communities with little political voice in low- and middle-income countries (LMICs) [13–15]. The term ‘unattended’ diseases, as used in the Global Goals for Sustainable Development (SDG 3) [16], may better reflect the lack of attention given to diseases affecting disadvantaged communities, as well as the ineffective, inappropriate or lack of application of available measures for disease control and prevention.

**Figure 1.**
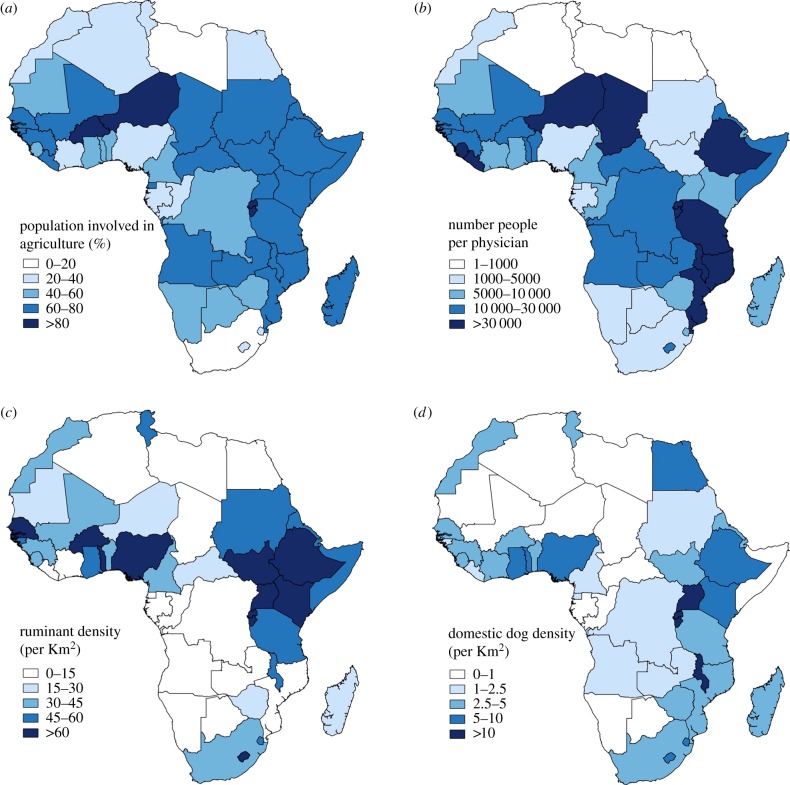
Maps showing African country-level data for: (*a*) percentage of population involved in agriculture—data from FAO [18]; (*b*) healthcare service provision shown by the number of people per physician—data from WHO [19]; (*c*) density of ruminant livestock (individuals km^−2^)—data from FAO [18]; (*d*) density of domestic dogs (dogs km^−2^)—data from Hampson *et al.* [2]. (Online version in colour.)

While people in all communities can be exposed to zoonotic infections, the greatest disease burden falls on the estimated one billion poor livestock keepers in Asia and Africa [1]. There are several reasons why it is the rural poor who are most vulnerable to exposure, infection and the downstream consequences of endemic zoonoses [13,14,17]: (i) because close contact with animals and traditional food consumption practices heighten exposure risks; (ii) because zoonotic diseases often affect livestock production, so it is people in poor livestock-dependent communities who are highly vulnerable to the impacts of zoonoses on livelihoods, food security and wellbeing; (iii) because the rural poor generally have limited access to high-quality human and animal health services for clinical care and treatment of illness; and (iv) because there are numerous social, political and economic issues that affect the ability of individuals to act in particular ways (individual agency), so the poor often have limited capacity to mitigate or manage disease risks. Figure 1 illustrates those countries in Africa, particularly in East and West Africa, where risks and vulnerabilities are likely to be particularly intense, characterized by areas where there is convergence of high livestock and domestic dog densities, a high proportion of the population engaged in agriculture and poor provision of health services.

## 2. A global rationale for prioritizing endemic zoonoses

Global health security is a shared responsibility but profound weaknesses in health systems currently limit the capacity of LMICs for effective healthcare provision, disease surveillance and outbreak response. Several international health initiatives address zoonoses, but many of these, including the International Health Regulations [20] and the Global Health Security Agenda (GHSA) [21], focus primarily on emerging zoonoses that threaten the broader global community.

The case has been made that a global surveillance system established for emerging zoonotic diseases could be readily improvised to address endemic diseases [22]. However, even with substantial investments made during outbreaks of highly pathogenic avian influenza, laboratory diagnostic capacity remains limited and concentrated in a few cities, and there remains a severe shortage of health professionals and workers, particularly in rural areas [23]. Thus, despite large-scale investments in surveillance for diseases such as highly pathogenic avian influenza, fewer than 20% of United Nations member states are able to effectively implement the International Health Regulations [24]. Once the immediate threat of an emerging disease has passed, and crisis funding and donor support removed, it may be difficult for governments in low-income countries to maintain support for the staff, skills and laboratory infrastructure needed to detect and respond to rare emerging disease outbreaks that are of little day-to-day concern to their populations.

To achieve effective global surveillance systems, we suggest that a complementary approach also needs to be considered—that is, a global surveillance and control system established to address endemic diseases that are acknowledged as priorities in developing countries can form the platform for a sustainable and effective surveillance system for early detection and response to emerging disease threats. The lessons of past disease outbreaks and response efforts show that the adaptation of existing networks and capacities to deal with new threats is crucial. This approach has recently been advocated in the regional meeting of Health and Agriculture Ministries in Latin America on the management of zoonose risks, with emerging, re-emerging and endemic zoonoses considered as linked priorities, and with integrated surveillance and coordinated governance key pillars of strengthening One Health capability [25]. We hypothesize that building systems to tackle endemic zoonotic challenges can provide a useful mechanism to build the core capacities that can then be adapted and built upon to achieve effective coordinated responses to future disease threats.

The engagement of several African countries in the GHSA zoonoses action package may provide a useful test of this hypothesis. While the focus of the GHSA is on preventing, detecting and responding to emerging zoonotic disease outbreaks that are of global concern, the GHSA zoonoses action package specifically advocates strengthening surveillance through One Health approaches and by focusing on the five zoonotic diseases or pathogens of ‘greatest public health concern’ [21]. For example, following a prioritization exercise that was conducted in Ethiopia, rabies, anthrax, brucellosis, leptospirosis and echinococcus were identified as the five zoonoses of greatest concern in Ethiopia, and recommendations were made for strengthening intersectoral surveillance and interventions against these diseases, with regular review to address new emerging zoonotic disease threats [26]. The implementation of these recommendations in Ethiopia should provide a useful indication as to whether and how strengthening of disease surveillance systems that address endemic zoonotic disease priorities will prepare the country to more effectively address emerging disease threats.

We have previously suggested that approaches focused on endemic zoonoses not only offer a pragmatic approach to overcoming existing barriers that limit global capacity for emerging disease surveillance, but also address inequalities in global health by delivering benefits to affected people in low-income countries [27]. This approach aligns closely with the recently established aims of the Global Goals for Sustainable Development within Goal 3. These include a commitment to accelerating progress not only in relation to diseases that are of widespread international concern (such as HIV/AIDS, tuberculosis and Ebola virus disease) but equally to those that affect disadvantaged communities, i.e. the neglected or unattended diseases affecting developing countries (Target 3.3) [16]. Goal 3 further combines a commitment to strengthening capacity of all countries, in particular developing countries, for early warning, risk reduction and management of national and global health risks (Target 3.d) with a target of achieving universal health coverage and access to affordable essential medicines and vaccines for all (Target 3.8) [16]. Within the Latin America region, One Health approaches that address both endemic and emerging disease are now being advocated specifically in the context of achieving these global goals for sustainable development [25].

## 3. Community trust, engagement and empowerment

In tackling zoonotic disease threats, technical capabilities such as diagnostic tests have often been prioritized over organizational capacities, such as communication, trust building, political advocacy and leadership, that are critical for improving institutions and systems [28]. However, among several important lessons learned, the West Africa epidemic of Ebola virus disease highlighted the critical need for enhancing community trust, engagement and ownership [23,29]. Intersectoral governance mechanisms have often been established in response to crises to address specific risks. However, trust between stakeholders and communities to support and build effective health systems cannot be spontaneously generated. Trust is built and is founded on experience, and can be developed through planned and regular interactions of all stakeholders, including representatives of heterogeneous communities, to address endemic zoonotic risks. Sustained investments and efforts to manage endemic zoonoses which deliver public health and livestock production benefits, train people and engage with communities can create a platform upon which relationships and trust can be built for more coordinated emergency responses.

## 4. Power, politics and agency

A major problem in tackling many zoonoses is that diagnosis and clinical management of human cases can be challenging, costly and usually requires access to reliable and high-quality medical services. While improvements in diagnosis and clinical management are urgently needed, we also need to understand the specific, economic, political and social forces that constrain the agency of individuals to act in particular ways. One Health can provide important understanding of the systemic reasons why some individuals are more affected by zoonoses than others because of its focus on animal–human interactions and on identifying which humans are interacting with which animals under what conditions [30]. These previously under-recognised vulnerabilities to disease intersect with socio-economic power relations and other structural factors, which in turn constrain health-seeking behaviour. For example, a study of women's experiences when seeking treatment for fever, which was carried out in a deprived urban community in Tanzania, highlighted the sense of helplessness experienced by these women when negotiating the health system:

I have a fear of the payment, and not of the sickness. Treatment exists, good treatment that will cure quickly, but you worry you will not be able to find the money in time because a fever does not wait for you. You worry if you will be able to find the money to get treatment before the patient dies [31, p. 128].

Where deaths occurred, ‘user fees’, ‘poverty’ and ‘inequality’ would not appear on death certificates, but a social autopsy would inevitably reveal these as conspiring factors in such fatalities [31]. Other problems that were frequently reported included waiting for treatment, while other, less-disadvantaged people ‘queue-jumped’, and having to provide bribes to be seen by doctors. These women's experiences demonstrate that poverty is more than just a lack of resource to make better decisions; they also point to the ways in which the sense of themselves as capable agents was being eroded. It did not matter what action these women took, the system appeared always to set them aside while others were privileged.

In considering the well-recognized links between poverty and ill-health, Farmer [32,33] emphasizes the importance of seeing beyond a causality that results from either failures in individual or household knowledge and behaviour, or from uncritical, relativistic understandings of cultural difference:

Exaggeration of patient agency is particularly marked in the biomedical literature, in part because of medicine's celebrated focus on individual patients, which inevitably desocializes [33, p. 258].

Farmer argues that sickness among the poor can be understood as the result of ‘structural violence’, historically and geographically specific, economic, political and social forces which work to constrain the agency of individuals to act in particular ways [32,33]. Whether this refers to government or international agency, policy, gender relations or capitalist relations playing out on a variety of scales, such structural processes create conditions that empower certain individuals with agency, while limiting the sense of capacity for others. Equitable delivery of health services is clearly necessary to reduce the toll of structural violence.

In a noteworthy case study, Chami *et al*. [34] discuss how power structures have played out in mass drug administration (MDA) campaigns in Uganda, impacting not only people seeking care for acute illness but also on delivery of anthelminthic drugs against schistosomiasis and soil-transmitted helminths. Socioeconomic status and minority group affiliation were key determinants of who received drugs and who did not, with people of low socioeconomic status and those in minority tribes or religions having less access to drugs. The contrast was particularly marked across households with or without members in the current or former village government. The failure to recognize how poverty—shaped by structural, political, socio-cultural and economic factors—influences both zoonotic disease risk and corresponding health-seeking behaviour is further reinforced through the focus on clinical management of ill health.

## 5. Protecting vulnerable populations and tackling inequalities through One Health interventions

The Commission on Social Determinants of Health established by the World Health Organization in 2005 has the remit of promoting health equity and of fostering a global movement to achieve it [35]. A driving principle of the work of this commission is that ‘No country or region should have to live with levels of ill-health that are avoidable’ [36]. Although treatments are available for several endemic zoonoses, it is the disadvantaged and poor who are being ‘left behind’ when approaches rely on clinical management. However, many endemic zoonoses are entirely or largely preventable through One Health measures targeted at animal or environmental reservoirs and infection sources and these preventive approaches offer several advantages.

One Health interventions that effectively reduce the force of infection from the animal or environmental reservoir convey benefits to all who are epidemiologically connected to the source of infection without regard to socioeconomic status—the benefit cannot be ‘purchased’ or socially distorted to the detriment of the poor. Social factors will always impact on the accessibility of healthcare but interventions targeted to prevent zoonoses at source will help buffer the impacts of these social drivers of inequality in healthcare provision, particularly in rural communities in Africa, where 83% of people are not covered by essential health services [37] and where people are also at greatest risk from endemic zoonoses [1,14].

In public health, the principle that preventive measures can be more effective and equitable than relying on treatments or cures is well accepted. When implemented on a national level, vaccination has been one of the most equitable low-cost, high-impact public health measures, saving millions of lives annually in LMICs [38]. However, this principle has been embraced less when the public health intervention is targeted at the animal population, even when the intervention has shown to be feasible and cost-effective, as in the case of mass vaccination of dogs against rabies, discussed below. One Health provides a useful framework for broadening the scope of potential interventions that might be considered by public health agencies, particularly in rural communities. In the case of livestock-mediated zoonoses, further advantages of One Health interventions relate to the added benefits to human health and livelihoods generated through improvements to livestock health and productivity.

One Health interventions advocate close intersectoral cooperation, interdisciplinary expertise and the involvement and empowerment (and not simply, engagement) of multiple stakeholders [39]. Thus, although few examples of such comprehensive One Health implementation exist, they not only provide a useful framework for addressing the types of disease problem that involve complex interactions between people, animals and the environment, but also offer a way to develop and implement more effective, appropriate and acceptable strategies for disease control and prevention.

## 6. Rabies as a case study

A clear example of the value of One Health interventions is provided by approaches to the prevention of human rabies deaths. Human rabies is 100% preventable through two complementary measures: first, post-exposure prophylaxis (PEP), which involves administration of rabies immunoglobulin and a multi-dose course of rabies vaccination to people bitten by suspected rabid animals; second, mass vaccination of animal reservoirs (primarily domestic dogs, the reservoir in the vast majority of human cases), which reduces the risk of human exposure and can ultimately result in rabies virus elimination.

While PEP is highly effective in preventing deaths in people exposed to the virus, many challenges remain for poor people in remote, rural communities in accessing and completing PEP regimens [40,41]. Delays in receiving the first dose of vaccine can all result in fatal outcomes, and occur as a result of vaccine being available only in larger clinics, a generally poor transport infrastructure, and/or the need to raise cash to cover medical and transport costs. In rural Tanzania, where most people still live on less than US$2 per day, patients would need to spend over US$100 to complete WHO recommended PEP schedules [41]. These challenges are compounded by intermittent vaccine shortages, particularly at rural health facilities, which further contribute to delays in patients receiving PEP and their inability to complete full schedules [42].

The realities of current approaches to the management of rabies exposures are revealed by data on the outcome of rabies exposures in 844 people from detailed contact-tracing studies [40] conducted in Tanzania from 1996 to 2016. Eighty individuals were recorded to have died from rabies, 71 (89%) of whom had not received any PEP at all, and none of the remaining nine had received a full course. The critical need for prompt PEP administration is shown by four rabies victims who developed rabies despite a delay of only 1 day in receiving the first vaccine dose. The poignancy of these preventable deaths is highlighted by two of these cases where patients had reported immediately to health facilities, but faced health system delays in receiving the first vaccine dose, with fatal consequences.

**Figure 2.**
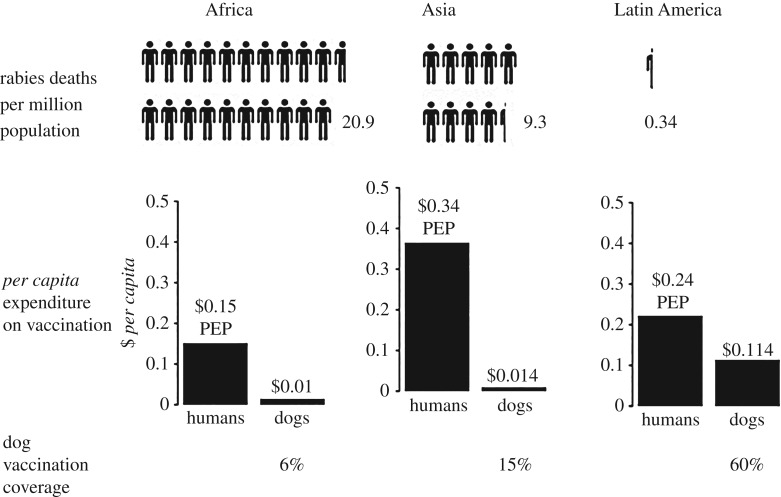
Scheme illustrating the relative expenditure on human rabies PEP and mass vaccination of dogs in relation to dog vaccination coverage and the incidence of human rabies deaths in Africa, Asia and Latin America. Data from Hampson *et al*. [2].

Rabies also illustrates the critical importance of connecting human and animal health services in the implementation of cost-effective preventive measures. Human deaths can be prevented by a combination of prompt administration of PEP and mass vaccination of domestic dog reservoirs, but the relative levels of investment in these two arms of prevention are often mismatched. In Asia, for example, the incidence of human rabies cases is much higher than in Latin America, despite the elevated levels of *per capita* expenditure on human PEP provision in Asia (figure 2). Here, even though health sector expenditure on PEP is very high (with US$ 1.3 billion of direct costs spent annually on PEP in Asia), poor people are still dying from rabies due to lack of access to health services with PEP. Conversely, many doses of PEP are given to animal-bite victims who will have had no rabies exposure, often in relatively affluent urban areas. By contrast, measures to prevent rabies at source (i.e. through mass dog vaccination) protect both the rich and the poor, casting a wider ‘safety net’ than can be achieved by focusing on management of human exposures alone and cost considerably less. In Latin America, for example, even modest investments in mass dog vaccination (US$ 61 million per year, representing approximately 20% total expenditure on rabies prevention) [2] have been highly effective in preventing human rabies deaths, with the region on the brink of eliminating dog rabies as a human health problem [43].

## 7. Zoonoses causing human febrile illness

The benefits of One Health preventive measures extend well beyond rabies and are likely to apply to many livestock zoonoses, including anthrax, brucellosis, leptospirosis, toxoplasmosis, Q fever and Rift Valley fever, all diseases for which livestock vaccines are available. As is the case with rabies, livestock zoonoses occur widely in LMICs, but remain largely ‘invisible’, with frequent mismanagement of animal and human cases contributing to a vicious cycle of ill-health and poverty [4].

Several livestock zoonoses—brucellosis, leptospirosis, Q fever and rickettsioses—which are known to occur across Africa [44–48] have been identified as causes of febrile illness in both adults and children in East and West Africa and southeast Asia [6,7,9,11,49–54]. Illnesses caused by these zoonotic pathogens are often difficult to diagnose and are frequently misdiagnosed: the clinical symptoms and signs are often non-specific, and easily mistaken for malaria [6]; laboratory confirmatory tests are rarely available; healthcare provider awareness is generally low [5]; and patients often present at hospital only in the later stages of infection when diagnostic confirmation is much more challenging. As malaria transmission declines in many parts of Africa [51,55], the importance of zoonoses as causes of non-malaria febrile illness is becoming more apparent. For example, in a study involving 870 patients hospitalized with fever in northern Tanzania [6], bacterial zoonoses, which were not initially considered by clinicians in any cases, were confirmed as a cause of disease in 26.2% of cases. Malaria, although clinically diagnosed in the majority of cases (60.7%), was confirmed as the cause of fever in only very few (1.6%). While the relative contribution of different zoonoses to the aetiology of febrile illness varies by location, livestock-associated zoonoses have been consistently identified in several recent studies from East Africa. For example, leptospirosis and Q fever were confirmed in a small number of cases of systemic febrile infections in children in one outpatient study in Tanzania [51], and in a much higher proportion of febrile children in an outpatient study from a different region, also in Tanzania (with 11.6% patients diagnosed with presumptive acute leptospirosis, 13% with confirmed leptospirosis and 22.4% with presumptive brucellosis) [11].

For most zoonoses that have been implicated as significant causes of human febrile illness, reliable point-of-care diagnostic tests are not available to support clinical management of cases. While these tests are urgently needed, challenges are still likely to remain in application and interpretation of diagnostic test results. Many patients will experience delays in reaching health facilities [56] and even with the best tests, pathogen detection in clinical samples can be very difficult. A further concern relates to the apparently high levels of prior antimicrobial use among febrile patients presenting to health facilities, which may compromise capacity to detect some bacterial infections. In an analysis of patients presenting with acute leptospirosis in Tanzania, urinary antibacterial activity was detected by bioassay in 31 (64.6%) of 48 cases, and is likely to have contributed to the inability of PCR diagnostic tests to detect infection in either plasma or urine samples at the time of presentation [57].

While point-of-care serological tests are available for some zoonoses, such as brucellosis, many challenges remain in the interpretation of results, including problems associated with the poor performance of tests for diagnosing acute cases and the high levels of background exposure in many livestock-keeping communities, which complicates interpretation of results based on a single serological test [58,59]. The likelihood of misdiagnosis and over-diagnosis of brucellosis is emerging as a clear problem in some areas, particularly in pastoral communities, and may well contribute to over-prescription and over-use of antimicrobials in these areas.

## 8. Designing One Health interventions: the example of livestock vaccination

Although a wide range of potential interventions may be considered based on a One Health rationale, particular opportunities exist in relation to livestock vaccination strategies in Africa, not only for improving livestock productivity and livelihoods, but also for tackling the preventable human health burden of endemic zoonoses. Several vaccines exist and are available to prevent many of the widely occurring livestock-associated zoonoses, and although they have been adopted in more intensive farming systems in high- and middle-income countries, they are not widely used in Africa or other low-income contexts.

Livestock vaccination against brucellosis and leptospirosis has been effective in reducing the burden of disease in many parts of the world. Vaccination of sheep and goats is the mainstay of current national brucellosis control and elimination strategies that are being implemented across Eastern Europe and Central Asia [60], with substantial declines in small ruminant and human disease documented in Azerbaijan, Kyrgyzstan, Macedonia and vaccinated regions of Tajikistan [60]. In some countries where small ruminant vaccination has been implemented, bovine brucellosis has also been reduced (e.g. Bosnia and Herzegovina, Kyrgyzstan), indicating that a greater proportion of bovine brucellosis is attributable to *Brucella melitensis* infection than is commonly considered [60]. These outcomes are consistent with findings from Tanzania that implicate sheep and goats as the likely source of infection in both people and cattle [61]. In Mongolia, small ruminant vaccination has also been shown to be a highly cost-effective strategy when considering both human health benefits and livestock production gains [62].

Vaccination of cattle against leptospirosis is widely practiced in intensive cattle dairy farms in Europe, North America and Australasia to reduce the reproductive and milk production losses associated with *Leptospira* infection, providing economic benefits to the farmer [63]. However, little information is available as to the broader population-level impacts of human health benefits of livestock vaccination schemes, and this remains an important gap that needs to be addressed [64].

Although there are few formal livestock vaccination programmes against Q fever, vaccination of cattle has been shown to be effective in reducing abortions and bacterial shedding in cattle under experimental conditions in Europe, and the long-term effectiveness of different vaccination strategies in an intensive dairy herd has been explored through modelling approaches [65]. During the 2007–2010 Q-fever outbreak in the Netherlands, livestock vaccination was also used for the first time with the objective of reducing the number of human Q-fever cases [66].

While existing vaccines offer great potential for preventing a wide range of livestock zoonoses that are widespread across Africa and other LMICs, many questions still remain as to the design, evaluation and implementation of such interventions. Livestock vaccination interventions cannot be rolled out uncritically and there may be occasions where vaccination efforts could have negative impacts. This might occur, for example, if vaccination results in an increase in the average age of an infection and might cause more reproductive losses in sexually mature animals than in situations where individuals become exposed and acquire immunity at a younger age.

Human and veterinary vaccination programmes are evaluated in very different ways [67]. While methods are now well established for the development and evaluation of complex human health interventions [68], these approaches have rarely been employed for interventions focusing on animal populations, even when these are designed to have human health benefits and outcomes. The added dimension of human–animal interaction inevitably adds complexity to the design of One Health interventions, and this is further compounded by the complex epidemiology of most livestock zoonoses—a taxonomically diverse group of pathogens, often comprising multiple subtypes of related pathogens circulating in multiple host species and with multiple modes of transmission.

Key questions in the design of vaccination programmes relate to the identification of target populations and individuals for vaccination, age at vaccination, vaccination coverage targets, understanding perceptions and potential barriers to uptake of vaccination, and sustainability of vaccination programmes to deliver public health benefits. For example, uptake may be compromised if animals identified for vaccination under epidemiologically optimal strategies are not the same as those considered by owners as the most valuable (e.g. in terms of traction power, food provision or social value). Household-level decisions will also need to be made as to how to balance potential costs for healthcare and treatment against recurring costs of preventive measures. These decisions will be particularly difficult for interventions directed at disease syndromes with multiple aetiologies, such as febrile illness, when the links between animal infection and human disease may be poorly appreciated, and the impact of any single intervention will only reduce a proportion of the overall burden of the disease syndrome (box 1).

Box 1. **Brucellosis—a case study.**

Brucellosis, caused by several species of the genus *Brucella*, is a debilitating human disease and cause of substantial livestock productivity losses globally, particularly in endemic countries [69]. Different *Brucella* species are associated with different animal hosts and people are most commonly infected through contact with diseased animals or consumption of infected animal products [70]. Brucellosis has been effectively controlled in many countries through strategies that include livestock vaccination, test-and-slaughter and sanitation measures. However, these have yet to be widely deployed in many African countries or other LMICs. Live attenuated vaccines including *Brucella abortus* strains (S19 and RB51) in cattle and *B. melitensis* Rev 1 in sheep and goats have been used successfully, but none of the current vaccines can protect all host species against all *Brucella* species. Safety issues also remain, and little is known about the use of vaccines in ‘non-target’ species, despite the possibility of cross-species infection and cross-protection [71].

In many low-income settings, questions also remain as to the design of optimal vaccination strategies, including whether to vaccinate against *B. abortus*, *B. melitensis*, other *Brucella* species or multiple species, which host species to vaccinate and whether specific age and sex subgroups should be targeted. In East Africa, both *B. abortus* and *B. melitensis* are present, but the degree to which their epidemiology overlaps in mixed livestock systems is largely unknown. Although emphasis is increasingly being placed on private sector incentives, government involvement is likely to be necessary for successful control of brucellosis in endemic areas [72]. National programmes can be highly cost-effective when the costs and benefits for human health and livestock sectors are both considered [62], but achieving an appropriate distribution of investments and benefits across sectors and among stakeholders remains challenging.

A key issue for brucellosis, and many endemic zoonoses, relates to poor ‘appreciability’ of diseases, particularly those that cause non-specific disease syndromes, such as human febrile illness and livestock abortion. This is likely to affect the investments that individuals, communities and stakeholders are willing to make in interventions. In contrast to mass dog vaccination strategies against rabies, which result in rapid, tangible benefits that are recognized by communities (i.e. the distinctive and visible cases of rabies disappear), control of brucellosis may be less readily appreciable. Even if successful, cases of fever in people and livestock abortion will still occur, and the interval between the intervention and any impact is likely to be prolonged. Understanding and clear communication of the multi-factorial causes of common disease syndromes (as experienced by affected communities) is crucial to ensure that expectations of any intervention are realistic and the likely outcomes clearly communicated.

As for all preventive measures, One Health interventions will be most equitable when delivered at population level. This poses several challenges for interventions based on livestock vaccination, which tend to focus on economic benefits from improved livestock productivity. National campaigns and government investment in livestock disease control are usually limited to notifiable and transboundary livestock diseases, and most countries lack coordinated programmes for large-scale control of endemic diseases, including many zoonoses [67]. Even where attention is focused on control of animal diseases for improving public health and rural development, current policies emphasize private sector involvement, with attention being given to innovative financing mechanisms, such as Development Impact Bonds [73], as well as product and market development to facilitate and support uptake by smallholder farmers [74]. However, if One Health interventions have to be sustained only or largely by direct payments from animal owners, they are unlikely to help overcome existing inequalities that disadvantage the rural poor in relation to affordability or access to health services. To achieve successful control of many zoonoses (e.g. brucellosis) [70] and the population-level benefits that would address existing health inequalities, careful consideration clearly needs to be given to the balance of private and public investment, to ensure sustainability and equity and to justify the use of scarce public resources.

## 9. Implementation and scaling-up of animal vaccination strategies

To achieve population-level benefits, particular care will be needed to avoid suboptimal vaccination outcomes, both in terms of vaccination coverage and completeness. For effective control of rabies, for example, it is increasingly clear that sustained, contiguous and large-scale programmes are needed, as even small pockets of low vaccination coverage (involving less than 0.5% of the dog population) can significantly hamper progress [75]. Even where the cost-effectiveness of One Health strategies has been demonstrated, for example, in relation to mass rabies vaccination of dogs in Africa [76,77], sheep and goat vaccination against brucellosis in Mongolia [62] and control of human African trypanosomiasis in Uganda [13], barriers remain to scaling-up.

Increasing attention is being paid to opportunities for integrating platforms for delivery of health interventions, with international health agencies focusing on the importance of strengthening health systems to achieve equitable delivery of health services, rather than developing multiple vertical programmes for specific diseases [78]. It is recognized that establishing independent delivery efforts for specific diseases can lead to fragmentation, and that coordination across sectors is needed to strengthen health systems. In Tanzania, these ideas are being explored through a programme investigating whether the reach and cost-effectiveness of MDA targeting soil-transmitted helminths in children and mass vaccination of domestic dogs against rabies can be improved through integration. Adopting One Health approaches, the project aims to break down traditional barriers that exist between veterinary and human health interventions, and build on the community trust achieved through the provision of a common good (effective control of rabies) in order to achieve cost savings, develop synergies and improve the effectiveness of both interventions. Further opportunities to establish co-delivery across a range of health interventions exist, not only for integrating control measures against several different zoonoses [73], but also for cross-linkages with public health interventions, such as water, sanitation and hygiene (WASH) programmes, and joint delivery of public health and veterinary services, particularly in remote or nomadic rural communities [79].

## 10. Conclusion

We make the case that One Health interventions that deploy existing tools, such as animal vaccination, to mitigate the impacts of endemic zoonoses can provide a pragmatic approach to achieving multiple objectives for global health. One Health interventions have the potential to overcome some of the existing social, political and economic challenges that constrain healthcare delivery in disadvantaged communities in Africa and deliver more equitable and cost-effective control of the endemic and neglected zoonoses that currently exert a substantial, although poorly recognized, burden of human and animal disease. Further, these approaches have the potential to enhance capacity for responding to emerging zoonotic disease threats through improved cross-sectoral collaboration, community engagement and the building of trust that comes through a shared sense of common good.

## References

[1] Grace D et al. 2012 *Mapping of poverty and likely zoonoses hotspots*. Zoonoses Project 4. Report to the UK Department for International Development. Nairobi, Kenya: International Livestock Research Institute. See http://hdl.handle.net/10568/21161.

[2] Hampson K et al. 2015 Estimating the global burden of endemic canine rabies. PLoS Negl. Trop. Dis. 9, e0003709 (10.1371/journal.pntd.0003709) 25881058 PMC4400070

[3] Costa F et al. 2015 Global morbidity and mortality of leptospirosis: a systematic review. PLoS Negl. Trop. Dis. 9, e0003898 (10.1371/journal.pntd.0003898) 26379143 PMC4574773

[4] Halliday JE, Allan KJ, Ekwem D, Cleaveland S, Kazwala RR, Crump JA 2015 Endemic zoonoses in the tropics: a public health problem hiding in plain sight. Vet. Rec. 176, 220–225. (10.1136/vr.h798) 25722334 PMC4350138

[5] Zhang HL et al. 2016 Mixed methods survey of zoonotic disease awareness and practice among animal and human healthcare providers in Moshi, Tanzania. PLoS Negl. Trop. Dis. 10, e0004476 (10.1371/journal.pntd.0004476) 26943334 PMC4778930

[6] Crump JA et al. 2013 Etiology of severe non-malaria febrile illness in Northern Tanzania: a prospective cohort study. PloS Negl. Trop. Dis. 7, e2324 (10.1371/journal.pntd.0002324) 23875053 PMC3715424

[7] Suttinont C et al. 2006 Causes of acute, undifferentiated, febrile illness in rural Thailand: results of a prospective observational study. Ann. Trop. Med. Parasitol. 100, 363–370. (10.1179/136485906X112158) 16762116

[8] Suputtamongkol Y, Rolain JM, Losuwanaruk K, Niwatayakul K, Suttinont C, Chierakul W, Pimda K, Raoult D 2003 Q fever in Thailand. Emerg. Infect. Dis. 9, 1186–1187. (10.3201/eid0909.030086) 14531384 PMC3016777

[9] Mayxay M et al. 2013 Causes of non-malarial fever in Laos: a prospective study. Lancet Glob. Health 1, e46–e54. (10.1016/s2214-109x(13)70008-1) 24748368 PMC3986032

[10] Susilawati TN, McBride WJ 2014 Acute undifferentiated fever in Asia: a review of the literature. Southeast Asian J. Trop. Med. Public Health 45, 719–726. 24974656

[11] Chipwaza B, Mhamphi GG, Ngatunga SD, Selemani M, Amuri M, Mugasa JP, Gwakisa PS 2015 Prevalence of bacterial febrile illnesses in children in Kilosa district, Tanzania. PLoS Negl. Trop. Dis. 9, e0003750 (10.1371/journal.pntd.0003750) 25955522 PMC4425467

[12] Chikeka I, Dumler JS 2015 Neglected bacterial zoonoses. Clin. Microbiol. Infect. 21, 404–415. (10.1016/j.cmi.2015.04.022) 25964152 PMC4466158

[13] WHO. 2006 The control of neglected zoonotic diseases: a route to poverty alleviation. In *Report of a joint WHO/DfID-AHP Meeting, 20–21 September, 2005*. Geneva, Switzerland: World Health Organization. See http://www.who.int/zoonoses/Report_Sept06.pdf.

[14] Molyneux D et al. 2011 Zoonoses and marginalised infectious diseases of poverty: where do we stand? Parasit. Vectors 4, 106 (10.1186/1756-3305-4-106) 21672216 PMC3128850

[15] Seimenis A 2012 Zoonoses and poverty—a long road to the alleviation of suffering. Vet. Ital. 48, 5–13. 22484998

[16] WHO. 2015 Chapter 5: Infectious diseases. In *Health in 2015: from MDGs, millennium development goals to SDGs, sustainable development goals*, pp. 101–130. Geneva, Switzerland: World Health Organization. See http://www.who.int/gho/publications/mdgs-sdgs/MDGs-SDGs2015_chapter5.pdf?ua=1.

[17] Maudlin I, Eisler MC, Welburn SC 2009 Neglected and endemic zoonoses. Phil. Trans. R. Soc. B 364, 2777–2787. (10.1098/rstb.2009.0067) 19687045 PMC2865085

[18] FAO. 2014 Global livestock production and health atlas. Rome, Italy: FAO. See http://kids.fao.org/glipha/.

[19] WHO. 2010 World Health Statistics, 2010. Geneva, Switzerland. See http://www.who.int/whosis/whostat/EN_WHS10_Full.pdf?ua=1.

[20] WHO. 2008 *International Health Regulations (2005), 2nd edn*. Geneva, Switzerland: World Health Organization. See http://www.who.int/ihr/9789241596664/en/.

[21] Global Health Security Agenda. 2016 *Global Health Security Agenda*. See https://ghsagenda.org.

[22] The World Bank. 2010 *People, pathogens and our planet. Volume 1: Towards a One Health approach for controlling zoonotic diseases*. Washington, DC: The International Bank for Reconstruction and Development/The World Bank. See http://siteresources.worldbank.org/INTARD/Resources/PPP_Web.pdf.

[23] United Nations. 2016 UN High-Level Panel on the Global Response to Health Crises. Protecting humanity from future health crises. New York, NY: United Nations.

[24] Burkle FM Jr 2015 Global health security demands a strong international health regulations treaty and leadership from a highly resourced World Health Organization. Disaster Med. Public Health Prep. 9, 568–580. (10.1017/dmp.2015.26) 25690046

[25] Pan American Health Organization. 2016 *Inter-American Ministerial Meeting on Health and Agriculture: One Health and the Sustainable Development Goals, Asuncion, Paraguay, 21–22 July 2016*. Washington, DC: Pan American Health Organization.

[26] Pieracci EG et al. 2016 Prioritizing zoonotic diseases in Ethiopia using a one health approach. One Health 2, 131–135. (10.1016/j.onehlt.206.09.001) 28220151 PMC5315415

[27] Halliday J et al. 2012 Bringing together emerging and endemic zoonoses surveillance: shared challenges and a common solution. Phil. Trans. R. Soc. B 367, 2872–2880. (10.1098/rstb.2011.0362) 22966142 PMC3427560

[28] Swanson RC et al. 2015 Strengthening health systems in low-income countries by enhancing organizational capacities and improving institutions. Global. Health 11, 5 (10.1186/s12992-015-0090-3) 25890069 PMC4340278

[29] WHO. 2014 *High level meeting on building resilient systems for health in Ebola-affected countries, 10–11 December 2014*. Geneva, Switzerland: World Health Organization. See http://www.who.int/mediacentre/events/meetings/2014/ebola-health-systems/en/

[30] Dzingirai V et al. 2016 Zoonotic diseases: who gets sick, and why? Explorations from Africa. Crit. Public Health 27, 97–110. (10.1080/09581596.2016.1187260)

[31] Laurie EW 2015 The embodied politics of health in Dar Es Salaam, Tanzania. PhD thesis, University of Glasgow, UK.

[32] Farmer P 2005 Pathologies of power: health, human rights, and the new war on the poor. Berkeley, CA: University of California Press.

[33] Farmer P 2001 Infections and inequalities: the modern plagues. Berkeley, CA: University of California Press.

[34] Chami GF, Kontoleon AA, Bulte E, Fenwick A, Kabatereine NB, Tukahebwa EM, Dunne DW 2016 Profiling nonrecipients of mass drug administration for schistosomiasis and hookworm infections: a comprehensive analysis of praziquantel and albendazole coverage in community-directed treatment in Uganda. Clin. Infect. Dis. 62, 200–207. (10.1093/cid/civ829) 26409064 PMC4690482

[35] WHO. 2008 *Closing the gap in a generation. Health equity through action on the social determinants of health*. Commission on Social Determinants of Health, Final Report. See http://www.who.int/social_determinants/thecommission/finalreport/en/.

[36] Marmot M 2007 Achieving health equity: from root causes to fair outcomes. Lancet 370, 1153–1163. (10.1016/s0140-6736(07)61385-3) 17905168

[37] Scheil-Adlung X (ed.) 2015 *Global evidence on inequities in rural health protection. New data on rural deficits in health coverage*. ESS document no. 47. Geneva, Switzerland: International Labour Office.

[38] Miller MA, Sentz JT 2006 Vaccine-preventable diseases. In Disease and mortality in Sub-Saharan Africa (eds Jamison DT, Feachem RG, Makgoba MW, Bos ER, Baingana FK, Hofman KJ, Rogo KO), 2nd edn, chapter 12. Washington, DC: The International Bank for Reconstruction and Development/The World Bank See https://www.ncbi.nlm.nih.gov/books/NBK2284/. 21290641

[39] Bardosh K 2016 One Health: science, politics and zoonotic disease in Africa. Abingdon, UK: Earthscan Routledge.

[40] Hampson K, Dobson A, Kaare M, Dushoff J, Magoto M, Sindoya E, Cleaveland S 2008 Rabies exposures, post-exposure prophylaxis and deaths in a region of endemic canine rabies. PLoS Negl. Trop. Dis. 2, e339 (10.1371/journal.pntd.0000339) 19030223 PMC2582685

[41] Sambo M, Cleaveland S, Ferguson H, Lembo T, Simon C, Urassa H, Hampson K 2013 The burden of rabies in Tanzania and its impact on local communities. PLoS Negl. Trop. Dis. 7, e2510 (10.1371/journal.pntd.0002510) 24244767 PMC3820724

[42] Mtema Z et al. 2016 Mobile phones as surveillance tools: implementing and evaluating a large-scale intersectoral surveillance system for rabies in Tanzania. PLoS Med. 13, e1002002 (10.1371/journal.pmed.1002002) 27070315 PMC4829224

[43] Vigilato MAN, Clavijo A, Knobl T, Silva HMT, Cosivi O, Schneider MC, Leanes LF, Belotto AJ, Espinal MA 2013 Progress towards eliminating canine rabies: policies and perspectives from Latin America and the Caribbean. Phil. Trans. R. Soc. B 368, 20120143 (10.1098/rstb.2012.0143) 23798691 PMC3720041

[44] Dean AS, Crump L, Greter H, Schelling E, Zinsstag J 2012 Global burden of human brucellosis: a systematic review of disease frequency. PLoS Negl. Trop. Dis. 6, e1865 (10.1371/journal.pntd.0001865) 23145195 PMC3493380

[45] Rubach MP, Halliday JEB, Cleaveland S, Crump JA 2013 Brucellosis in low-income and middle-income countries. Curr. Opin. Infect. Dis. 26, 404–412. (10.1097/QCO.0b013e3283638104) 23963260 PMC3888775

[46] de Vries SG, Visser BJ, Nagel IM, Goris MG, Hartskeerl RA, Grobusch MP 2014 Leptospirosis in sub-Saharan Africa: a systematic review. Int. J. Infect. Dis. 28, 47–64. (10.1016/j.ijid.2014.06.013) 25197035

[47] Vanderburg S, Rubach MP, Halliday JE, Cleaveland S, Reddy EA, Crump JA 2014 Epidemiology of *Coxiella burnetii* infection in Africa: a OneHealth systematic review. PLoS Negl. Trop. Dis. 8, e2787 (10.1371/journal.pntd.0002787) 24722554 PMC3983093

[48] Allan KJ, Biggs HM, Halliday JE, Kazwala RR, Maro VP, Cleaveland S, Crump JA 2015 Epidemiology of leptospirosis in Africa: a systematic review of a neglected zoonosis and a paradigm for ‘One Health’ in Africa. PLoS Negl. Trop. Dis. 9, e0003899 (10.1371/journal.pntd.0003899) 26368568 PMC4569256

[49] Maina AN, Farris CM, Odhiambo A, Jiang J, Laktabai J, Armstrong J, Holland T, Richards AL, O'Meara WP 2016 Q fever, scrub typhus, and rickettsial diseases in children, Kenya, 2011–2012. Emerg Infect. Dis. 22, 883 (10.3201/eid2205.150953) 27088502 PMC4861507

[50] Maina AN et al. 2012 *Rickettsia felis* infection in febrile patients, western Kenya, 2007–2010. Emerg. Infect. Dis. 18, 328–331. (10.3201/eid1802.111372) 22304807 PMC3310467

[51] D'Acremont V et al. 2014 Beyond malaria—causes of fever in outpatient Tanzanian children. N. Engl. J. Med. 370, 809–817. (10.1056/NEJMoa1214482) 24571753

[52] Njeru J, Henning K, Pletz MW, Heller R, Neubauer H 2016 Q fever is an old and neglected zoonotic disease in Kenya: a systematic review. BMC Public Health 16, 297 (10.1186/s12889-016-2929-9) 27048480 PMC4822290

[53] Njeru J et al. 2016 Human brucellosis in febrile patients seeking treatment at remote hospitals, northeastern Kenya, 2014–2015. Emerg. Infect. Dis. 22, 2160–2164. (10.3201/eid2212.160285) 27662463 PMC5189133

[54] Ki-Zerbo GA, Tall F, Nagalo K, Ledru E, Durand G, Patey O 2000 Rickettsiosis and Q fever in pyretic patients hospitalized at the Bobo-Dioulasso Hospital (Burkina Faso). Med. Mal. Infect. 30, 270–274. (10.1016/S0399-077X(00)89140-4)

[55] Bhatt S 2015 The effect of malaria control on *Plasmodium falciparum* in Africa between 2000 and 2015. Nature 526, 207–211. (10.1038/nature15535) 26375008 PMC4820050

[56] Odiit M, Shaw A, Welburn SC, Fevre EM, Coleman PG, McDermott JJ 2004 Assessing the patterns of health-seeking behaviour and awareness among sleeping-sickness patients in eastern Uganda. Ann. Trop. Med. Parasitol. 98, 339–348. (10.1179/000349804225003389) 15228715

[57] Allan KJ 2016 Leptospirosis in northern Tanzania: investigating the role of rodents and ruminant livestock in a neglected public health problem. PhD thesis, University of Glasgow, UK. See http://theses.gla.ac.uk/view/creators/Allan=3AKathryn_J=2E=3A=3A.html.

[58] Ducrotoy M et al. 2015 Brucellosis in sub-Saharan Africa: current challenges for management, diagnosis and control. Acta Trop. 165, 179–193. (10.1016/j.actatropica.2015.10.023) 26551794

[59] de Glanville WA et al. 2017 Poor performance of the rapid test for human brucellosis in health facilities in Kenya. PLoS Negl. Trop. Dis. 11, e0005508 (10.1371/journal.pntd.0005508) 28388625 PMC5413359

[60] FAO. 2015 Regional workshop on brucellosis control in Central Asia and Eastern Europe. *FAO Animal Production and Health Report No. 8*. Rome, Italy: Food and Agriculture Organization of the United Nations. See http://www.fao.org/3/a-i4387e.pdf.

[61] Viana M, Shirima GM, John KS, Fitzpatrick J, Kazwala RR, Buza JJ, Cleaveland S, Haydon DT, Halliday JE 2016 Integrating serological and genetic data to quantify cross-species transmission: brucellosis as a case study. Parasitology 143, 821–834. (10.1017/s0031182016000044) 26935267 PMC4873909

[62] Roth F, Zinsstag J, Orkhon D, Chimed-Ochir G, Hutton G, Cosivi O, Carrin G, Otte J 2003 Human health benefits from livestock vaccination for brucellosis: case study. Bull. World Health Organ. 81, 867–876. 14997239 PMC2572379

[63] Sergeant ES 1992 Leptospirosis vaccination in beef cattle: use of decision tree analysis. N. Z. Vet. J. 40, 62–65. (10.1080/00480169.1992.35699) 16031659

[64] Durski KN, Jancloes M, Chowdhary T, Bertherat E 2014 A global, multi-disciplinary, multi-sectorial initiative to combat leptospirosis: Global Leptospirosis Environmental Action Network (GLEAN). Int. J. Environ. Res. Public Health 11, 6000–6008. (10.3390/ijerph110606000) 24905245 PMC4078561

[65] Courcoul A, Hogerwerf L, Klinkenberg D, Nielen M, Vergu E, Beaudeau F 2011 Modelling effectiveness of herd level vaccination against Q fever in dairy cattle. Vet. Res. 42, 68 (10.1186/1297-9716-42-68) 21605376 PMC3125226

[66] Hogerwerf L, van den Brom R, Roest HIJ, Bouma A, Vellema P, Pieterse M, Dercksen D, Nielen M 2011 Reduction of *Coxiella burnetii* prevalence by vaccination of goats and sheep, the Netherlands. Emerg. Infect. Dis. 17, 379–386. (10.3201/eid1703.101157) 21392427 PMC3166012

[67] Knight-Jones TJ, Edmond K, Gubbins S, Paton DJ 2014 Veterinary and human vaccine evaluation methods. Proc. R. Soc. B 281, 20132839 (10.1098/rspb.2013.2839) PMC4043076 24741009

[68] Craig P, Dieppe P, Macintyre S, Michie S, Nazareth I, Petticrew M 2008 Developing and evaluating complex interventions: the new Medical Research Council Guidance. BMJ 337, a1655 (10.1136/bmj.a1655) 18824488 PMC2769032

[69] Pappas G, Papadimitriou P, Akritidis N, Christou L, Tsianos EV 2006 The new global map of human brucellosis. Lancet Infect. Dis. 6, 91–99. (10.1016/s1473-3099(06)70382-6) 16439329

[70] WHO, FAO, OIE. 2003 Brucellosis in humans and animals. Geneva, Switzerland: World Health Organization. See http://www.who.int/csr/resources/publications/Brucellosis.pdf.

[71] Godfroid J, Kasbohrer A 2002 Brucellosis in the European Union and Norway at the turn of the twenty-first century. Vet. Microbiol. 90, 135–145. (10.1016/S0378-1135(02)00217-1) 12414139

[72] Avila-Calderon ED, Lopez-Merino A, Sriranganathan N, Boyle SM, Contreras-Rodriguez A 2013 A history of the development of *Brucella* vaccines. Biomed. Res. Int. 2013, 743509 (10.1155/2013/743509) 23862154 PMC3686056

[73] Welburn SC, Beange I, Ducrotoy MJ, Okello AL 2015 The neglected zoonoses—the case for integrated control and advocacy. Clin. Microbiol. Infect. 21, 433–443. (10.1016/j.cmi.2015.04.011) 25911990

[74] GALVmed. 2016 *Protecting livestock, improving human lives*. See https://www.galvmed.org/en/about-us/our-approach/.

[75] Townsend SE et al. 2013 Designing programs for eliminating canine rabies from islands: Bali, Indonesia as a case study. PloS Negl. Trop. Dis. 7, e2372 (10.1371/journal.pntd.0002372) 23991233 PMC3749988

[76] Zinsstag J, Durr S, Penny MA, Mindekem R, Roth F, Menendez Gonzalez S, Naissengar S, Hattendorf J 2009 Transmission dynamics and economics of rabies control in dogs and humans in an African city. Proc. Natl Acad. Sci. USA 106, 14 996–15 001. (10.1073/pnas.0904740106) PMC2728111 19706492

[77] Fitzpatrick MC, Hampson K, Cleaveland S, Mzimbiri I, Lankester F, Lembo T, Meyers LA, Paltiel AD, Galvani AP 2014 Cost-effectiveness of canine vaccination to prevent human rabies in rural Tanzania. Ann. Intern. Med. 160, 91–100. (10.7326/m13-0542) 24592494 PMC4084874

[78] Bill and Melinda Gates Foundation. *Integrated delivery strategy overview*. http://www.gatesfoundation.org/What-We-Do/Global-Development/Integrated-Delivery.

[79] Schelling E, Wyss K, Béchir M, Moto DD, Zinsstag J 2005 Synergy between public health and veterinary services to deliver human and animal health interventions in rural low income settings. BMJ 331, 1264–1267. (10.1136/bmj.331.7527.1264) 16308393 PMC1289333

